# Integrated approach of federated learning with transfer learning for classification and diagnosis of brain tumor

**DOI:** 10.1186/s12880-024-01261-0

**Published:** 2024-05-15

**Authors:** Eid Albalawi, Mahesh T.R., Arastu Thakur, V. Vinoth Kumar, Muskan Gupta, Surbhi Bhatia Khan, Ahlam Almusharraf

**Affiliations:** 1 Department of Computer science, College of Computer Science and Information Technology, King faisal University, 31982 Hofuf, Saudi Arabia; 2 Department of Computer Science and Engineering, Faculty of Engineering and Technology, JAIN (Deemed-to-be University), 562112 Bangalore, India; 3 School of Computer Science Engineering and Information Systems, Vellore Institute of Technology, 632014 Vellore, India; 4 School of Science, Engineering and environment, University of Salford, M5 4WT Manchester, UK; 5 Department of Electrical and Computer Engineering, Lebanese American University, Byblos, Lebanon, Lebanon; 6 Department of Business Administration, College of Business and Administration, Princess Nourah bint Abdulrahman University, P.O. Box 84428, Riyadh, 11671 Riyadh, Saudi Arabia

**Keywords:** Brain tumor classification, MRI imaging, Convolutional neural networks, Federated learning, VGG16, Medical image analysis

## Abstract

Brain tumor classification using MRI images is a crucial yet challenging task in medical imaging. Accurate diagnosis is vital for effective treatment planning but is often hindered by the complex nature of tumor morphology and variations in imaging. Traditional methodologies primarily rely on manual interpretation of MRI images, supplemented by conventional machine learning techniques. These approaches often lack the robustness and scalability needed for precise and automated tumor classification. The major limitations include a high degree of manual intervention, potential for human error, limited ability to handle large datasets, and lack of generalizability to diverse tumor types and imaging conditions.To address these challenges, we propose a federated learning-based deep learning model that leverages the power of Convolutional Neural Networks (CNN) for automated and accurate brain tumor classification. This innovative approach not only emphasizes the use of a modified VGG16 architecture optimized for brain MRI images but also highlights the significance of federated learning and transfer learning in the medical imaging domain. Federated learning enables decentralized model training across multiple clients without compromising data privacy, addressing the critical need for confidentiality in medical data handling. This model architecture benefits from the transfer learning technique by utilizing a pre-trained CNN, which significantly enhances its ability to classify brain tumors accurately by leveraging knowledge gained from vast and diverse datasets.Our model is trained on a diverse dataset combining figshare, SARTAJ, and Br35H datasets, employing a federated learning approach for decentralized, privacy-preserving model training. The adoption of transfer learning further bolsters the model’s performance, making it adept at handling the intricate variations in MRI images associated with different types of brain tumors. The model demonstrates high precision (0.99 for glioma, 0.95 for meningioma, 1.00 for no tumor, and 0.98 for pituitary), recall, and F1-scores in classification, outperforming existing methods. The overall accuracy stands at 98%, showcasing the model’s efficacy in classifying various tumor types accurately, thus highlighting the transformative potential of federated learning and transfer learning in enhancing brain tumor classification using MRI images.

## Introduction

Brain tumors pose intricate challenges due to their location in the delicate structure of the human brain. These abnormal masses of cells, which can be either benign or malignant, present a wide spectrum of complexities that extend beyond their classification. Understanding these complexities is crucial in comprehending the gravity of the condition and the intricacies of treatment.

Benign tumors, though noncancerous, can still cause significant issues depending on their location and size. They may exert pressure on the brain, leading to symptoms such as headaches, seizures, or neurological deficits. However, these tumors typically have well-defined borders and tend to grow slower than malignant tumors. Surgical removal might offer a curative option for these tumors, although their location within critical brain regions might limit the feasibility of complete resection without causing damage to essential brain structures.

In contrast, malignant brain tumors, also known as brain cancer, exhibit more aggressive behavior. They grow rapidly and infiltrate surrounding healthy brain tissue, making complete surgical removal challenging. The most common malignant primary brain tumor in adults is glioblastoma multiforme, notorious for its aggressive nature and resistance to treatment. Its diffuse nature within the brain makes it challenging to eradicate entirely, leading to a high recurrence rate despite aggressive treatment approaches involving surgery, radiation, and chemotherapy.

The diversity of brain tumors further complicates treatment strategies. There are distinct types of tumors, such as gliomas, meningiomas, pituitary adenomas, and medulloblastomas, each with their unique characteristics and challenges. For instance, some tumors originate from the brain tissue itself, while others may develop from surrounding structures or metastasize from cancers elsewhere in the body. This diversity demands tailored approaches for accurate diagnosis, prognosis, and treatment planning.

The skull serves as an unyielding shield, guarding the brain against external forces. However, this rigid structure becomes a hindrance when faced with internal growth, whether benign or malignant. Brain tumors, regardless of their nature, can pose severe challenges due to the limited space within the skull. Their presence often leads to heightened intracranial pressure, a condition that can culminate in brain damage or life-threatening situations.

The World Health Organization (WHO) adopts a systematic classification system for brain tumors, aiming to categorize them based on their type, level of malignancy, and grade. This categorization is pivotal in guiding the treatment approach and understanding the prognosis associated with each tumor type.

The skull’s rigidity means that any growth within this confined space can trigger a cascade of issues. Even benign tumors, while not cancerous, can exert substantial pressure on the brain as they grow. Their expansion within the limited confines of the skull can lead to a rise in intracranial pressure, which, in turn, might cause symptoms ranging from persistent headaches to nausea, vomiting, seizures, and even neurological deficits.

Malignant tumors, on the other hand, present a graver concern. Their aggressive nature, characterized by rapid growth and invasive tendencies, exacerbates the challenges posed by limited intracranial space. As these tumors progress, they infiltrate and displace healthy brain tissue, amplifying the elevation of intracranial pressure. This situation can quickly escalate, causing severe neurological impairment or life-threatening consequences if not managed promptly and effectively.

The WHO classification system for brain tumors is a vital tool in understanding the diverse landscape of these conditions. It categorizes tumors into several types based on their cellular origin, characteristics, and behavior. Moreover, it differentiates between grades, reflecting the level of malignancy and the tumor’s aggressiveness.

For instance, gliomas, a type of tumor originating from glial cells, encompass a spectrum ranging from low-grade (such as astrocytoma’s and oligodendrogliomas) to high-grade tumors like glioblastoma multiforme (GBM), known for their aggressive behavior. Meningiomas, arising from the meninges, are typically categorized as benign tumors, but depending on their location and growth pattern, they too can exert pressure on the brain and cause significant issues.

The WHO grading system further stratifies tumors based on their histopathological features, aiding clinicians in determining the prognosis and guiding treatment decisions. Grade I and II tumors are considered low-grade, often growing slowly, and possessing more defined borders, while Grade III and IV tumors represent high-grade malignancies, exhibiting rapid growth and infiltrative tendencies.

Magnetic Resonance Imaging (MRI) stands as a cornerstone in diagnosing brain tumors due to its ability to offer highly detailed images of the brain’s anatomy. However, interpreting these images accurately to diagnose and classify brain tumors poses a complex challenge. Traditionally, this task has relied on the expertise of radiologists, yet this manual interpretation is time-consuming, subjective, and susceptible to human error, especially in intricate cases or when managed by less experienced personnel.

The emergence of machine learning, particularly deep learning techniques, has revolutionized medical image analysis, presenting novel prospects for brain tumor diagnosis. Convolutional Neural Networks (CNNs), a type of deep learning algorithm, have exhibited remarkable potential in precisely categorizing images, including those from medical imaging. Their adeptness in learning intricate patterns and features from vast datasets renders them suitable for tasks like brain tumor classification. The types of Brain Tumors are discussed in Table 1.

The contemporary methodologies for brain tumor classification harness deep learning by training CNN models on extensive datasets comprising MRI images. These models are trained to discern and identify patterns and features associated with several types of brain tumors. Despite notable advancements, challenges persist in terms of data privacy, model generalization, and the demand for substantial, annotated datasets.

**Table 1:** Types of brain tumors

Tumor Type	Origin	Grade/Severity	Symptoms
Gliomas (arising from glial cells)	Brain tissue	Varies (I-IV)	Headaches, seizures, vision problems, weakness, personality changes
Meningioma	Membranes surrounding the brain	Usually, benign	Headaches, seizures, vision problems, numbness, weakness
Schwannoma	Nerve cells	Usually, benign	Hearing loss, tinnitus, dizziness, facial weakness
Pituitary Adenoma	Pituitary gland	Varies (benign, aggressive)	Vision problems, headaches, fatigue, excessive thirst, milk production (galactorrhea)
Medulloblastoma	Embryonic cells	Malignant	Headaches, nausea, vomiting, balance problems, difficulty walking
Craniopharyngioma	Near the pituitary gland	Usually, benign	Vision problems, headaches, fatigue, hormonal imbalances
Metastatic Brain Tumors	Spread from other cancers	Any grade	Varies depending on primary cancer

MRI’s unparalleled ability to produce high-resolution images of the brain enables detailed visualization of tumors, providing crucial information for diagnosis and treatment planning. However, the process of analyzing these images manually relies heavily on radiologists’ expertise, leading to subjectivity and potential errors. Moreover, interpreting complex MRI images to differentiate between various tumor types demands a profound understanding of subtle nuances that might not always be evident to the human eye.

The integration of deep learning techniques, especially CNNs, has shown immense promise in revolutionizing the interpretation of MRI images for brain tumor diagnosis. These algorithms can autonomously learn intricate patterns and features within images, potentially augmenting the accuracy and efficiency of tumor classification.

CNNs function by utilizing multiple layers to detect hierarchical patterns within images. They learn from large volumes of labeled data, gradually enhancing their ability to recognize specific features associated with diverse types of brain tumors. This learning process involves the extraction of features at various levels of abstraction, enabling the network to discern subtle variations indicative of distinct tumor characteristics.

Despite the considerable progress made with CNNs, challenges persist within this domain. Data privacy remains a concern due to the sensitive nature of medical imaging data. Annotated datasets, crucial for training deep learning models, are often limited in size and accessibility due to privacy regulations and the labor-intensive nature of labeling medical images.

Furthermore, ensuring the generalizability of these models beyond the datasets they were trained on remains a significant challenge. Models trained on specific datasets might encounter difficulties when applied to new, unseen data or when faced with variations in imaging techniques or equipment.

Efforts to address these challenges include the development of privacy-preserving techniques that enable model training without compromising patient data confidentiality. Transfer learning, a method where pre-trained models are fine-tuned with smaller datasets, offers a potential solution for mitigating the need for vast amounts of annotated data. Additionally, collaborations between healthcare institutions for data sharing and the creation of standardized datasets could facilitate model training and validation across diverse populations [1]. 

The integration of deep learning in brain tumor classification using MRI images signifies a promising avenue in improving diagnostic accuracy and efficiency. As technology advances and methodologies evolve, the synergy between machine learning and medical imaging is poised to enhance our ability to detect, classify, and manage brain tumors, potentially transforming patient care and outcomes. However, addressing challenges related to data privacy, model generalization, and dataset availability will be crucial in realizing the full potential of these advancements in clinical practice [2]. 

To address these challenges, we propose a novel federated learning-based deep learning model for automated and accurate brain tumor classification. This innovative approach not only emphasizes the use of a modified VGG16 architecture optimized for brain MRI images but also highlights the significance of federated learning and transfer learning in the medical imaging domain. Federated learning enables decentralized model training across multiple clients without compromising data privacy, addressing the critical need for confidentiality in medical data handling. Additionally, transfer learning leverages a pre-trained CNN, enhancing the model’s ability to classify brain tumors accurately by leveraging knowledge gained from vast and diverse datasets.The Contribution of the Research Paper are:

- The primary objective of this research is to develop and validate a federated learning-based CNN model for the classification of brain tumors from MRI images.
- This model aims to enhance classification accuracy while addressing data privacy concerns, a significant step forward in medical imaging and diagnostics.

The subsequent sections of the research paper encompass vital facets crucial for a comprehensive study. The “Related Work” segment intricately surveys existing technologies, offering a detailed overview of prevailing methodologies. Following this, the “Materials and Methods” section elaborates on the dataset used, the CNN model architecture, and the innovative federated learning approach. The “Results” segment showcases empirical findings, spotlighting the model’s performance via metrics like accuracy, precision, recall, and F1-scores. Subsequently, the “Discussion” section conducts a thorough analysis, comparing outcomes with established methods, exploring implications, and addressing study limitations. The “Conclusion” succinctly summarizes key findings’ potential impacts on medical diagnostics and delineates avenues for future research. Lastly, the “References” compile all referenced scientific literature and data sources, ensuring academic integrity, and acknowledging scholarly contributions.

## Related work

The field of medical imaging, particularly the classification and diagnosis of brain tumors using MRI images, has seen significant advancements with the integration of machine learning and deep learning techniques. This section reviews related work in this domain, highlighting key methodologies, findings, and how they relate to our current research.

- **Traditional Image Analysis Techniques**: While pioneering, traditional image processing techniques such as edge detection and region-based segmentation have been limited by their reliance on manual intervention and the potential for subjective interpretations. These methods laid the groundwork for automated analysis but often fell short in handling the complex and varied morphology of brain tumors, necessitating the development of more sophisticated, automated systems.
- **Machine Learning Approaches**: The advent of machine learning brought about a significant improvement in automated classification with algorithms like Support Vector Machines (SVM) and Random Forests. However, these approaches required extensive feature engineering to capture the nuances of tumor morphology, a process that is both labor-intensive and potentially limiting in capturing the full complexity of the data. Moreover, classical machine learning methods sometimes struggled to manage the high-dimensional nature of MRI data effectively [3].
- **Deep Learning Developments**: Deep learning models diagnose by analyzing patterns in vast datasets during training, where they adjust internal parameters through iterative processes to minimize errors between predicted and actual diagnoses. Once trained, these models apply learned patterns to new data to make diagnoses. However, understanding the precise reasoning behind each diagnosis can be challenging as deep learning models often operate as “black boxes,” lacking transparent decision-making processes. Despite their impressive accuracy, efforts to enhance interpretability, such as attention mechanisms and saliency maps, aim to shed light on the features or patterns influencing the models’ diagnoses, thereby improving trust and understanding in their clinical applications [4].
- **Federated Learning in Medical Imaging**: Federated learning emerges as a promising solution to some of these challenges, especially in addressing data privacy and scarcity. By enabling models to be trained across multiple decentralized datasets, federated learning circumvents the need for data centralization, thus preserving privacy. However, one critical challenge in federated learning is the potential introduction of communication overhead between devices, which can impact its efficiency [5, 6].
- **Multi-Task Learning and Transfer Learning**: Some studies have explored multi-task learning and transfer learning to improve the efficiency and generalizability of models. These models, while delivering impressive performance, often lack transparency in their decision-making process, making it challenging for clinicians and researchers to understand how they arrive at diagnoses [7, 8].

Our research builds upon and extends these developments by proposing a federated learning-based deep learning model, utilizing a modified VGG16 architecture for the classification of brain tumors from MRI images. This approach not only addresses the limitations associated with traditional techniques, machine learning, and deep learning methods but also leverages the strengths of federated learning to offer a novel solution that prioritizes precision, efficiency, and data privacy. By comparing with the existing methodologies outlined in Table 2, our study contributes a unique perspective to the ongoing dialogue in this rapidly evolving field, highlighting the potential of federated learning to overcome some of the most pressing challenges in healthcare applications.

**Table 2:** Existing methodologies

Study	Accuracy	Summary
Pedada, Kameswara Rao, et al. [9]	93.40% and 92.20%	Use of U-Net Model for the segmentation on Brats 2017 and 2018 dataset.
Saeedi, Soheila, et al. [10]	96.47%	2D CNN employed with ensemble techniques of machine learning.
Mahmud, Md Ishtyaq, Muntasir Mamun, and Ahmed Abdelgawad. [11]	93.3%	Redefined CNN Model with modified classification.
Wang, Nathan, et al. [12]	94.90%	Deep CNN on OCT Images.
Prakash, R. Meena, et al. [13]	97.39%	Hyperparameter tuning of dense net.
Senan, Ebrahim Mohammed, et al. [14]	95.10%	Alexnet + SVM
Haq, Amin ul, et al. [15]	97.40%	CNN with Transfer Learning
Rasool, Mohammed, et al. [16]	98.1%	GoogleNet along with SVM as classifier
Khan, Abdul Hannan, et al. [17]	94.84%	Hierarchical Deep Learning-Based Brain Tumor (HDL2BT) classification
Gaur, Loveleen, et al. [18]	94.64%	CNN with Gaussian Noise
Vidyarthi, Ankit, et al. [19]	95.86%	CNN with NN Classifier
Lamrani, Driss, et al. [20]	96%	CNN with Enhanced Classifiers

By comparing with these related studies, our research contributes to the ongoing dialogue in this rapidly evolving field, offering a novel approach that balances the need for precision, efficiency, and data security in healthcare applications.

## Methodology

**Fig. 1:**
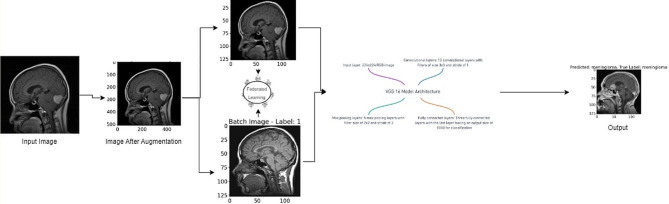
Proposed model

This research employs an advanced machine learning approach, combining Convolutional Neural Networks (CNNs) with a federated learning framework, to classify brain tumors using Magnetic Resonance Imaging (MRI) data. The methodology encompasses several key components: dataset preparation, model architecture design, federated learning implementation, training procedures, and evaluation metrics. The architecture of the proposed model has been given in Fig. 1.

### A. Dataset description and preparation

**Fig. 2:**
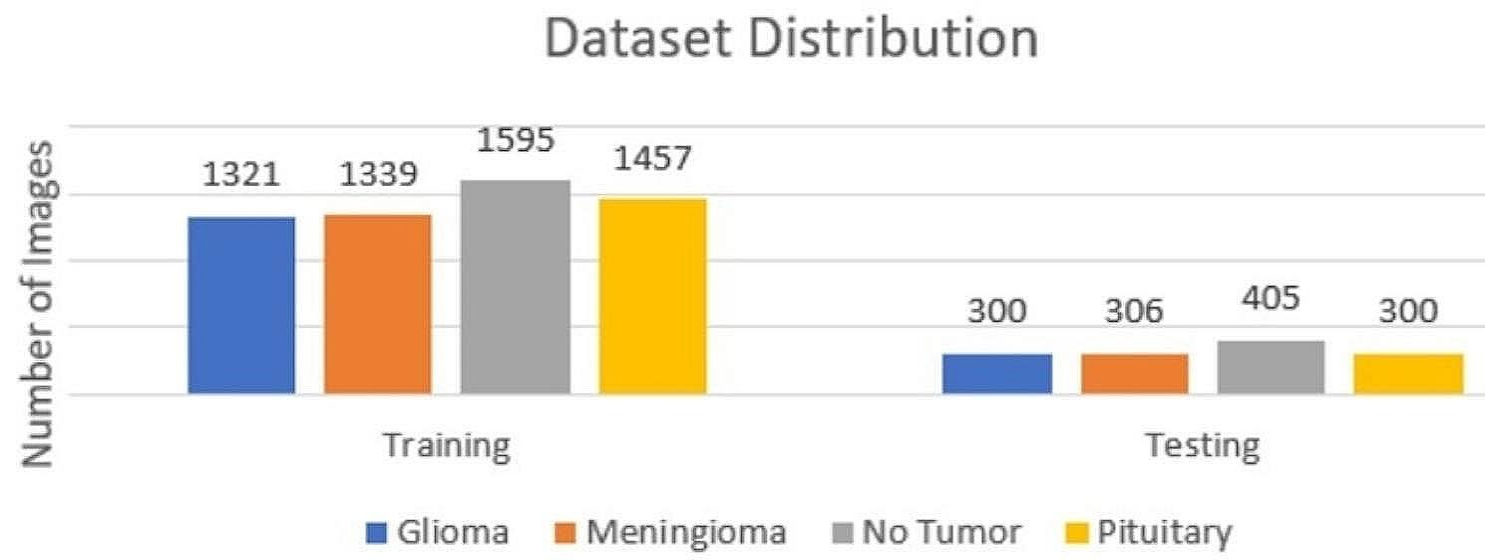
Dataset distribution

The research utilizes a comprehensive dataset comprising 7023 MRI images of the human brain, classified into four categories: glioma, meningioma, no tumor, and pituitary. This dataset is an amalgamation of data from three sources: figshare, the SARTAJ dataset, and Br35H. The images labeled as ‘no tumor’ were sourced from the Br35H dataset. Given concerns about the accuracy of glioma classification in the SARTAJ dataset, these images were replaced with those from figshare to ensure the integrity of the dataset. Table 3 shows the dataset description while Fig. 2 represents the dataset distribution.

**Table 3:** Dataset description

Type	Training	Testing
Glioma	1321	300
Meningioma	1339	306
No Tumor	1595	405
Pituitary	1457	300

**Fig. 3:**
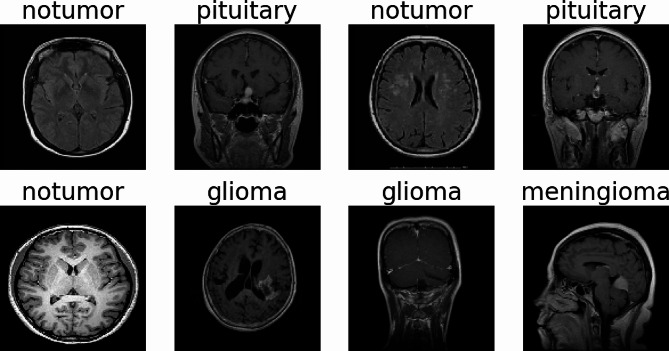
Sample images from the dataset

Each image in the dataset underwent a series of preprocessing steps. This included augmenting the images to improve the model’s ability to generalize and learn from a more diverse range of data representations. The augmentation techniques included adjusting brightness and contrast levels randomly within specified ranges. The images were then resized to a uniform size of 128 × 128 pixels to ensure consistency in input data for the model which can be seen in Fig. 3.

#### Image preprocessing techniques

##### Augmentation

**Fig. 4:**
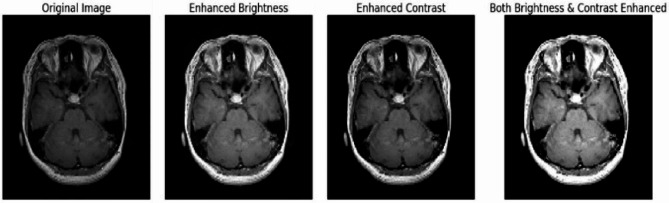
Images after augmentation

In a bid to counteract overfitting and bolster the model’s capacity to generalize beyond the training set, augmentation techniques were employed. These techniques introduced random modifications to the images’ brightness and contrast, mimicking the variability often encountered in real-world medical imaging scenarios. By infusing diversity into the dataset through augmentation, the model is better equipped to adapt to varying image characteristics during training, potentially enhancing its ability to make accurate predictions on unseen data. In Fig. 4 images after augmentation can be seen.

- **Normalization**: Following augmentation, a critical preprocessing step involved normalizing the images’ pixel values. This normalization procedure standardized the pixel intensity values, scaling them between 0 and 1. Such normalization is imperative for optimizing CNN model training. It aids in stabilizing and accelerating the training process by ensuring consistent data ranges across the entire dataset, thereby preventing certain features from disproportionately influencing the learning process.
- **Resizing**: Consistency in input dimensions is pivotal for Convolutional Neural Networks (CNNs) to effectively process images. Hence, all images underwent resizing to adhere to a uniform dimension of 128 × 128 pixels. This standardization ensures that the model receives inputs of a consistent size, facilitating uniform processing and enabling CNN to extract relevant features from the images consistently.

The preprocessing steps undertaken in this study—augmentation, normalization, and resizing—significantly contribute to enhancing the dataset’s quality and preparing the images for efficient utilization within the CNN model. Augmentation broadens the dataset’s variability, normalization standardizes pixel values for effective model training, and resizing ensures uniform input dimensions, collectively aiding in building a robust and reliable model for brain tumor classification.

Moreover, the strategic curation and refinement of the glioma class within the dataset underscore the study’s commitment to data quality and diversity, crucial factors influencing the CNN model’s performance and its potential applicability in real-world scenarios. This comprehensive dataset, augmented and preprocessed to optimize its utility for model training, lays a solid foundation for the subsequent phases of the study, enabling the development of an accurate and adaptable brain tumor classification model.

### B. Convolutional neural network (CNN) model architecture

The core of our methodology is the implementation of a Convolutional Neural Network (CNN) which can be seen in Eq. 1, specifically leveraging the VGG16 model architecture. The VGG16 model, a product of the Visual Graphics Group (VGG) at the University of Oxford, has garnered acclaim for its prowess in image recognition tasks. Its architecture comprises a sequence of convolutional layers, interspersed with max-pooling layers, culminating in a series of fully connected layers. Trained on the ImageNet dataset, it gained popularity due to its ability to discern intricate features within images, making it an ideal choice for various computer vision applications.

1 $$ \text{Output}=\text{ReLU}\left(\text{Convolution}\left(\text{Input},\text{Filters}\right)+\text{Bias}\right) $$

**Adaptation for brain tumor classification**: In this research, the VGG16 model which can be observed in Eq. 2 was adapted to cater specifically to the task of brain tumor classification. The original top layers, responsible for ImageNet’s classification into a thousand categories, were excised to tailor the architecture to the four distinct categories pertinent to brain tumors: glioma, meningioma, no tumor, and pituitary.

2 $$ \text{Output}=\text{ReLU}\left(\text{Conv}\left(X,W\right)+b\right)$$

### C. Model architecture

In preparing MRI images for compatibility with the VGG16 model, we implemented a series of preprocessing steps designed to optimize input data quality and consistency. This included resizing images to 224 × 224 pixels, the standard input size for VGG16, and applying a normalization process to scale pixel values to a range that matches the original VGG16 training data. To adapt the VGG16 architecture for the specialized task of brain tumor classification from MRI images, we introduced modifications that included fine-tuning the filter sizes in convolutional layers to better capture the nuances of MRI textures and adding additional dropout layers to prevent overfitting. Furthermore, our transfer learning strategy involved the utilization of pre-trained weights from the ImageNet dataset, leveraging the model’s existing feature extraction capabilities. This approach was complemented by fine-tuning the top layers of the model to align with our specific classification task, allowing the network to adjust to the distinct characteristics of brain tumors. The output layer of the model was reconfigured to support multi-class classification, replacing the original 1000-class output with a new layer designed to distinguish between four tumor categories: glioma, meningioma, no tumor, and pituitary. This layer employs a SoftMax activation function to output probabilities across these four categories, ensuring the model’s predictions align with the classification requirements of our study. Together, these tailored preprocessing steps, architectural modifications, and strategic application of transfer learning empower our CNN model to effectively classify brain tumors from MRI images with enhanced accuracy and generalizability.

- **Input Layer**: The model commences with an input layer designed to accept images of 128 × 128 pixels, embracing three color channels (Red, Green, Blue - RGB). This layer serves as the gateway for the images to traverse through the network.
- **Base Model**: The cornerstone of the architecture is the incorporation of the pre-trained VGG16 base model, shorn of its original top layers. Retaining the base layers while discarding the classification head allows the model to retain its proficiency in extracting intricate features from images while enabling its adaptation to the specific task at hand - brain tumor classification. These base layers come equipped with weights learned from the vast and diverse ImageNet dataset, serving as a valuable foundation for discerning pertinent features in our dataset.
- **Flattening Layer**: Following the convolutional layers, a flattening layer is introduced. This layer transforms the two-dimensional output from the last convolutional layer into a one-dimensional array, preparing the data for processing through subsequent fully connected layers.
- **Dense Layers**: The architecture incorporates several dense layers, often termed as fully connected layers, leveraging Rectified Linear Unit (ReLU) activation functions. These layers are pivotal in capturing and comprehending the intricate, non-linear relationships embedded within the data. By sequentially connecting these densely connected layers, the model can learn hierarchical representations of the input data, crucial for discerning complex patterns associated with brain tumor classification.
- **Dropout Layers**: To combat overfitting, a common concern in neural network models, dropout layers have been strategically incorporated. During the training phase, these layers randomly deactivate a fraction of input units, mitigating the reliance on specific neurons and preventing the network from overfitting to the training data. This regularization technique promotes the model’s ability to generalize well to unseen data.
- **Output Layer**: The architecture culminates in an output layer comprising a dense layer with a SoftMax activation function. This final layer is responsible for the classification task, assigning probabilities to each class (glioma, meningioma, no tumor, pituitary). The SoftMax function normalizes these probabilities, ensuring they sum up to one, thereby facilitating the categorization of the input image into one of the four distinct tumor categories based on the highest probability.
- **Fine-Tuning and Training**: The base layers of the VGG16 model were fine-tuned on our specific dataset. By making these layers trainable, the model could adapt and learn more relevant features related to brain tumor classification. This process involves updating the weights of these layers during the training phase, thereby tailoring the network’s representations to the intricacies and complexities inherent in our dataset.

The architecture of the CNN model, employing the modified VGG16 framework, embodies a structured hierarchy of layers meticulously designed for the intricate task of brain tumor classification. Leveraging the strengths of the VGG16 base model while customizing it to the specific requirements of our dataset, this architecture serves as a robust foundation for the subsequent phases of model training and evaluation, aiming to accurately classify brain tumors into distinct categories for enhanced clinical diagnosis and prognosis.

### D. Federated learning implementation

To address data privacy concerns and improve model robustness, we adopted a federated learning approach. In this approach, the model training is decentralized, with multiple clients (in this case, 10) training models on their subsets of data. This method ensures that sensitive medical data does not leave its original location, preserving patient privacy.

The federated learning process involved the following steps:

- **Client Selection**: The federated learning process initiates by randomly selecting a subset of clients for model training at each iteration or training round. In this scenario, approximately 50% of the total clients, totaling ten clients, were chosen for participation in each training round.
- **Local Training**: Upon selection, each client involved in the federated learning process receives a copy of the global model. This global model serves as the initial framework derived from the modified VGG16 architecture. Each client then proceeds to train this model locally on their respective datasets.
- The decentralized nature of federated learning allows each client to leverage their local dataset without transmitting any raw or identifiable patient data outside their environment. This local training process occurs autonomously at each client’s end, enabling them to iteratively update the model based on the unique characteristics and nuances within their dataset.
- **Model Aggregation**: Following the local training phase, the models from each client are aggregated to update the global model. This aggregation involves averaging the weights of the models obtained from the various clients. By amalgamating the locally trained models through weight averaging, the global model is iteratively refined and enhanced.

**Fig. 5:**
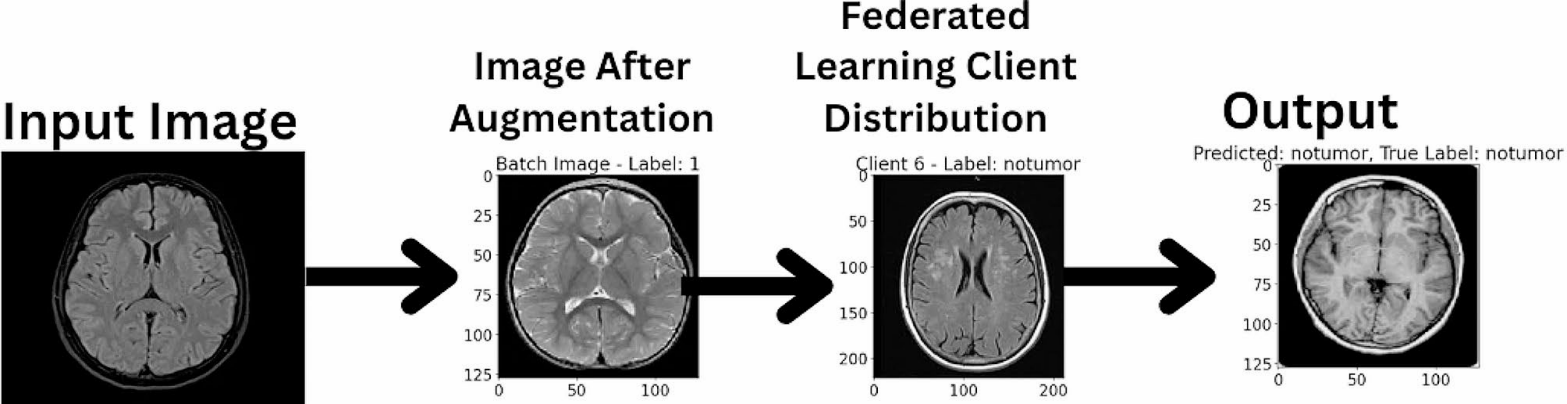
Different steps of image processing

Federated learning offers a robust and privacy-preserving paradigm for advancing brain tumor classification models. By distributing model training across multiple clients while maintaining data localization, this approach addresses data privacy concerns and augments model robustness through diverse datasets. The iterative refinement of the global model via model aggregation integrates insights from varied clinical contexts, paving the way for more accurate and adaptable brain tumor classification models with heightened privacy safeguards. The several steps that an image goes through during the classification can be observed in Fig. 5.

### E. Training and evaluation

The training process involved feeding the CNN model with batches of images, with a specified batch size and number of epochs. The model’s performance was evaluated using standard metrics, including accuracy, precision, recall, and F1-score.

In addressing the intricacies of federated learning within our brain tumor classification model, we acknowledge the inherent challenge of increased communication overhead that this distributed training approach entails. Federated learning necessitates frequent exchanges of model updates between the client and the central server, which can significantly strain network resources. To mitigate this overhead, we have employed strategies such as model compression techniques and sparsification, which reduce the size of the model updates being transmitted without compromising the integrity of the training process. A separate set of images, not used during the training phase, constituted the test dataset. This dataset was critical for assessing the model’s ability to generalize and accurately classify unseen data.

#### Training batches

**Fig. 6:**
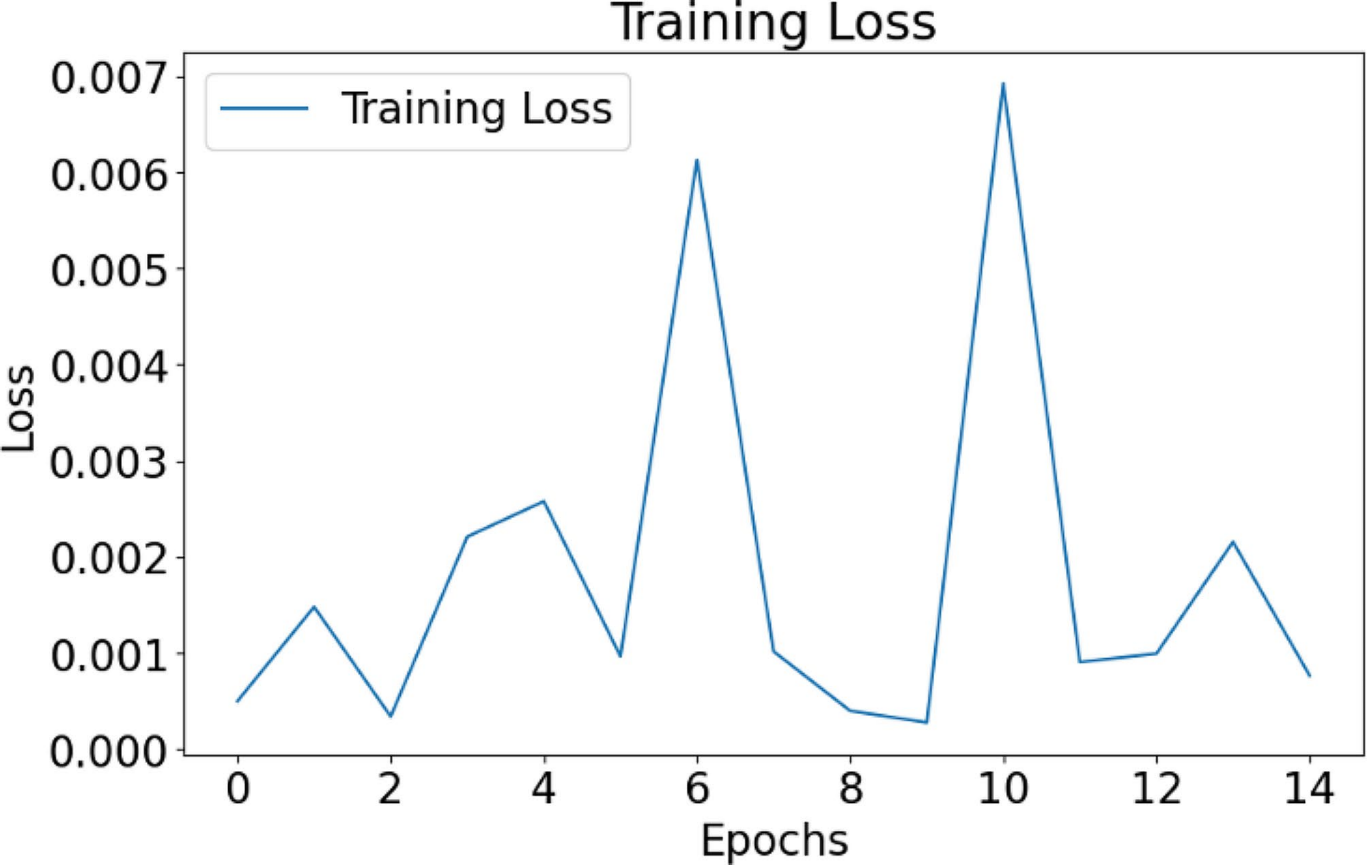
Training loss

- Image Processing and Augmentation: The training process begins with data processing and augmentation. The dataset is loaded and shuffled to ensure randomness, crucial for robust model training. Augmentation techniques, such as brightness and contrast adjustments, are applied to diversify the dataset and mitigate overfitting. Images are preprocessed to ensure uniformity in size and pixel values, enhancing the model’s ability to learn from various samples.
- Batch Processing for Efficient Learning: The model receives data in batches, a practice vital for efficient training. This strategy aids in managing memory resources and facilitates parallel processing, enabling the model to learn iteratively in manageable chunks rather than processing the entire dataset at once. The code segments demonstrate the creation of a data generator function that yields batches of images and labels, allowing the model to learn from a subset of data in each iteration. In Fig. 6 the training loss can be observed.

- **Validation set:** A crucial step in model development is the validation phase, where a subset of the dataset is reserved exclusively for validation purposes. This set acts as unseen data for the model, enabling the assessment of its generalization capabilities. Validation occurs iteratively during model training to monitor its performance on data it has not been trained on, safeguarding against overfitting, and ensuring the model’s ability to generalize to new, unseen samples.

#### Hyperparameter tuning:

- Optimization for Enhanced Performance: The success of the model hinges on optimal hyperparameter configuration. Hyperparameters, such as learning rate and dropout rates, significantly influence the model’s learning process. Tweaking these parameters is critical for achieving superior performance and preventing issues like underfitting or overfitting. The code illustrates setting these hyperparameters and their values for fine-tuning.
- Iterative Optimization: Hyperparameter tuning is an iterative process aimed at finding the most suitable values that optimize the model’s learning without compromising its ability to generalize. This iterative approach involves adjusting hyperparameters, training the model, evaluating its performance on the validation set, and iteratively refining the parameters to achieve the best possible model performance [17].

## Results and discussions

When assessing the effectiveness of a brain tumor classification model, a range of evaluation metrics is employed to gain comprehensive insights into its performance. These metrics serve as pivotal benchmarks to gauge the model’s accuracy, its ability to correctly identify different tumor types, and its overall efficacy in handling the classification task.

- **Accuracy**: Accuracy, which is calculated in Eq. 3, in the context of model evaluation, quantifies the proportion of correctly classified images out of the total number of images. While it offers a quick glimpse into the model’s overall performance, accuracy might not provide a complete picture, especially when dealing with imbalanced datasets. For instance, if one class dominates the dataset, the model might achieve high accuracy by simply predicting the majority class most of the time, neglecting the classification of minority classes [18].

1. TP = True Positives.
2. TN = True Negatives.
3. FP = False Positives.
4. FN = False Negatives.

3 $$ Accuracy=\frac{TP+TN}{TP+TN+FP+FN}$$

- **Precision**: Precision that is calculated in Eq. 4 delves deeper into the model’s performance by assessing the correctness of positive predictions. Specifically, it measures the ratio of correctly predicted positive instances (true positives) to the total number of instances predicted as positive (true positives + false positives). In the context of brain tumor classification, precision signifies how accurately the model identifies a specific tumor type when it makes a positive prediction for that class.

4 $$ Precision=\frac{TP}{TP+FP}$$

- **Recall**: Recall that is calculated using Eq. 5, also known as sensitivity or true positive rate, signifies the model’s ability to correctly identify all instances of a particular class among all the instances that belong to that class. It quantifies the ratio of correctly predicted positive instances (true positives) to the total number of actual positive instances (true positives + false negatives). In the context of brain tumor classification, recall emphasizes the model’s capability to detect and not miss instances of a particular tumor type.

5 $$ Recall=\frac{TP}{TP+FN}$$

- **F1-Score**: The F1-score that is calculated using Eq. 6 offers a harmonized measure that balances both precision and recall. It represents the harmonic means of precision and recall, providing a single metric to evaluate the model’s performance considering both false positives and false negatives. This metric is particularly useful when there is an imbalance between the classes or when both precision and recall are crucial for the classification task. In brain tumor classification, where each tumor type’s identification is vital, the F1-score becomes a critical metric to assess overall performance.

6 $$ F1\_score=2\times \frac{Precision\times Recall}{Precision+Recall}$$

In brain tumor classification, these metrics play a pivotal role in understanding the model’s ability to discern between different tumor types. For instance, precision would reveal how accurately the model identifies a specific tumor type among all the instances it classified as that type. Meanwhile, recall would emphasize how well the model detects all instances of a particular tumor type among the total instances of that type in the dataset.

In the next section, we will present and discuss the results obtained from this methodology.

The results obtained from the federated learning-based CNN model demonstrate its exceptional capability in accurately classifying brain tumors from MRI images. The model’s performance was assessed through various metrics including precision, recall, F1-score, and overall accuracy, supported by a detailed analysis using a confusion matrix.

### A. Model performance

**Fig. 7:**
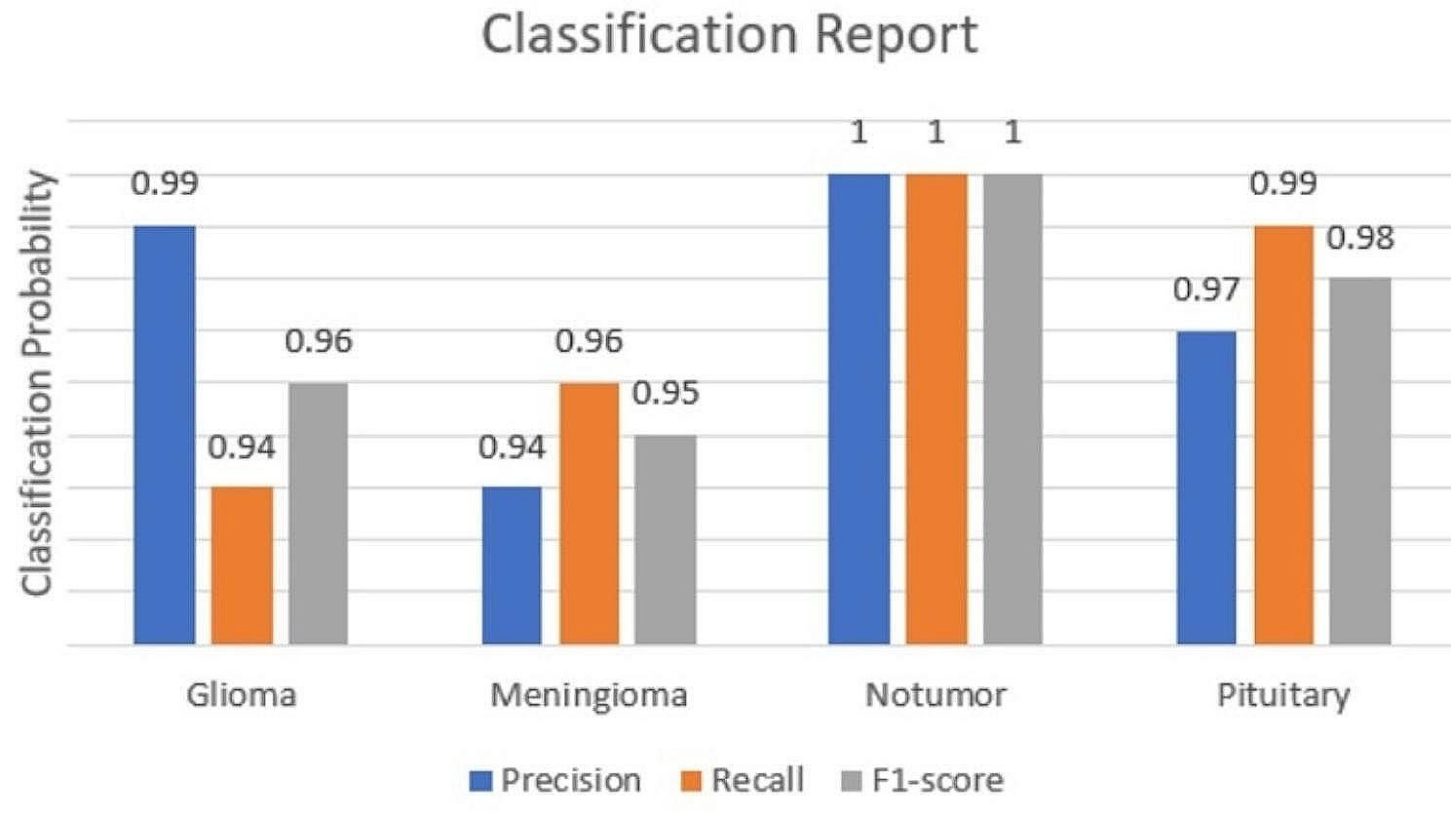
Classification report

The performance of the federated learning-based CNN model was rigorously evaluated using a comprehensive classification report, accuracy scores, and a confusion matrix. The following are the key findings:

- **Classification Report**: The model achieved remarkable precision, recall, and F1-scores across all four tumor classes. The results are given in the Table 4 followed by the Fig. 7:

**Table 4:** Classification report

Tumor	Precision	Recall	F1-score
Glioma	0.99	0.94	0.96
Meningioma	0.94	0.96	0.95
No tumor	1	1	1
Pituitary	0.97	0.99	0.98

**Fig. 8:**
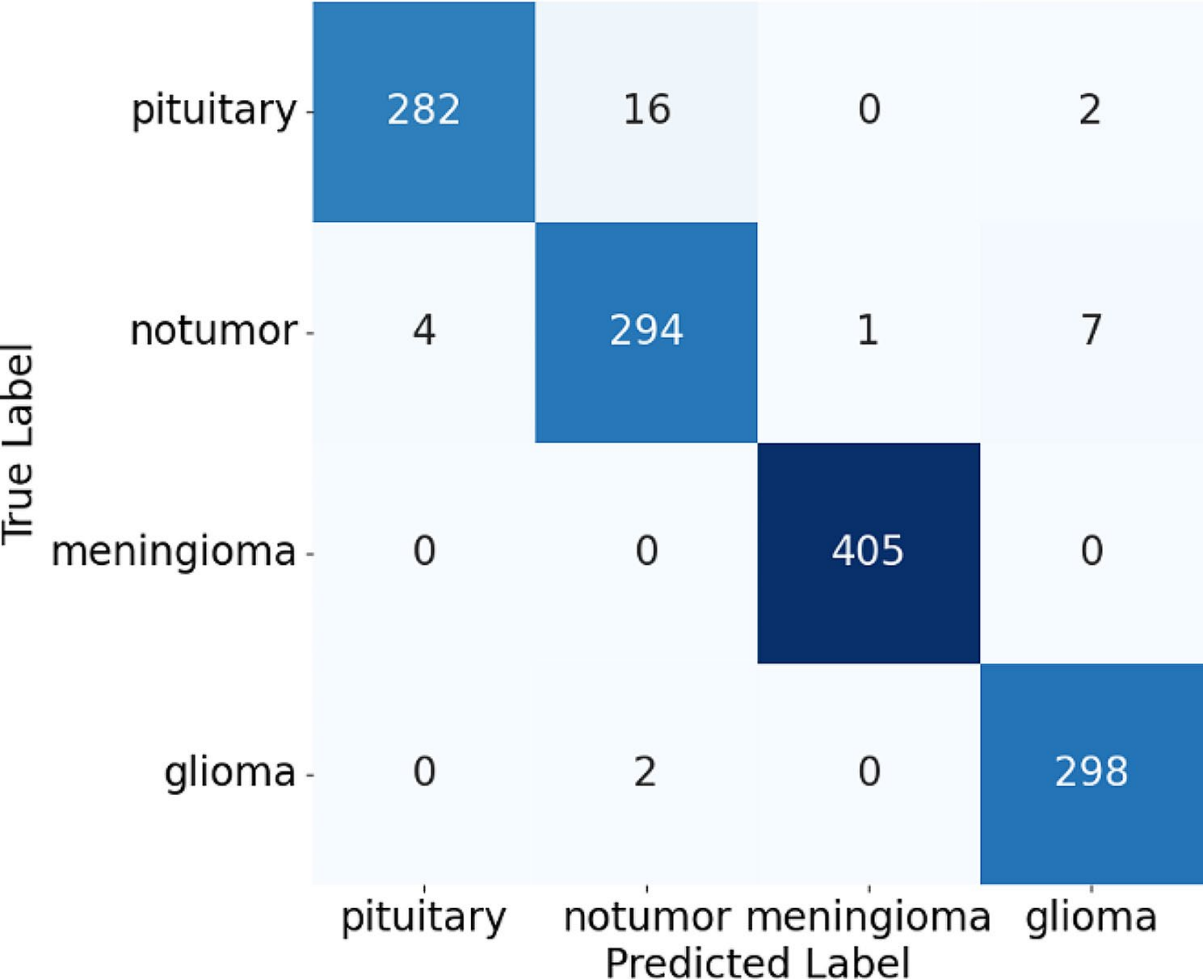
Confusion matrix

- **Accuracy Scores**: The model demonstrated an overall accuracy of 98%, indicating its effectiveness in correctly identifying the presence and type of brain tumors in MRI images.
- **Confusion Matrix**: A confusion matrix was generated to provide a visual representation of the model’s performance [23–24]. The matrix highlighted the true positives, false positives, true negatives, and false negatives for each tumor category. The high number of true positives and true negatives, along with the small number of false positives and false negatives, underscored the model’s accuracy which can be observed in Fig. 8.

### B. Analysis of results

The results from the model’s performance evaluation reveal several key insights:

- **High Precision and Recall**: The model’s high precision indicates a low rate of false positives, which is crucial in medical diagnostics to avoid unnecessary treatments. Similarly, the high recall scores suggest a low rate of false negatives, ensuring that the presence of tumors is accurately identified.
- **Effectiveness in Classifying Tumor Types**: The near-perfect F1-scores across all tumor types reflect the model’s exceptional ability to differentiate between glioma, meningioma, no tumor, and pituitary cases. This is particularly significant given the challenges associated with distinguishing between these tumor types using traditional methods.
- **Generalization Capability**: The high overall accuracy score demonstrates the model’s capability to generalize well across the diverse dataset. This suggests that the model can be reliably used in different clinical settings and with varying MRI image qualities.
- **Federated Learning Impact**: The implementation of federated learning contributed to the model’s robustness and accuracy. By training across multiple decentralized datasets, the model benefited from a wider variety of data, enhancing its ability to generalize and perform accurately on unseen data.

In conclusion, the proposed federated learning-based CNN model has proven highly effective in the classification of brain tumors using MRI images. Its high accuracy, precision, and recall make it a promising tool for aiding medical professionals in the diagnosis and treatment planning of brain tumors. The following section will discuss these results in the context of existing methodologies and explore their implications in the field of medical imaging.

### C. Comparison with existing methods

The federated learning-based CNN model marks a significant advancement over traditional and existing deep learning methods in brain tumor classification. Traditional approaches, reliant on manual interpretation of MRI images, are time-consuming and subject to human error. Even with the integration of conventional machine learning techniques, these methods often lack the robustness and adaptability required for precise tumor classification.

In contrast, existing deep learning methods, while more accurate than manual interpretation, frequently encounter challenges related to data privacy, model generalization, and dependency on large, well-annotated datasets. The proposed federated learning model addresses these concerns effectively. Its ability to achieve a high accuracy rate of 98%, with substantial precision and recall across all tumor types, sets it apart from previous deep learning approaches. Moreover, the decentralized nature of federated learning ensures data privacy and diversity, contributing to the model’s robust performance across various datasets. In the below table the comparison with the existing model is given in Table 5:

**Table 5:** Comparison with existing methodologies

Study	Accuracy	Technique
Pedada, Kameswara Rao, et al. [9]	93.40% and 92.20%	Use of U-Net Model for the segmentation on Brats 2017 and 2018 dataset.
Saeedi, Soheila, et al. [10]	96.47%	2D CNN employed with ensemble techniques of machine learning.
Mahmud, Md Ishtyaq, Muntasir Mamun, and Ahmed Abdelgawad. [11]	93.3%	Redefined CNN Model with modified classification.
Khan, Abdul Hannan, et al. [19]	94.84%	Hierarchical Deep Learning-Based Brain Tumor (HDL2BT) classification
Gaur, Loveleen, et al. [20]	94.64%	CNN with Gaussian Noise
Vidyarthi, Ankit, et al. [21]	95.86%	CNN with NN Classifier
Lamrani, Driss, et al. [22]	96%	CNN with Enhanced Classifiers
Islam, Moinul, et al. [23]	91.05%	Federated Learning
Alshammari, Abdulaziz. [24]	93.74%	VGG-16 with Integration of CNN
Proposed Model	98%	VGG with Federated Learning

### D. Challenges and limitations

Despite its successes, the study faces several challenges and limitations:

- **Data Biases and Diversity**: The model’s performance is contingent on the diversity and quality of the data on which it is trained. Biases in the dataset, such as overrepresentation of certain tumor types or imaging styles, could potentially skew the model’s learning and prediction accuracy [25].
- **Federated Learning Complexities**: While federated learning offers benefits in data privacy and diversity, it also introduces complexities in model training and aggregation. Ensuring consistent model performance across different clients with potentially non-IID (independently and identically distributed) data is a challenge.
- **Scalability and Computational Resources**: The scalability of the federated learning approach and the computational resources required for training and aggregating models across multiple clients are significant considerations, especially in resource-constrained setting [26].

The generalization of findings to diverse patient populations remains a limitation. The study’s results, while promising, require further validation in cohorts that encompass a wider range of demographic, geographic, and clinical characteristics. Such validation studies are essential to confirm the model’s applicability and effectiveness across different settings and populations, ensuring that the benefits of federated learning for brain tumor classification can be realized on a global scale.

### E. Future directions

To further enhance the model’s efficacy and applicability, several future directions are suggested:

- **Expanding Dataset Diversity**: Testing the model on a more diverse set of MRI images, including those from different demographics and with varying imaging conditions, would improve its robustness and generalizability.
- **Cross-Institutional Collaboration**: Implementing the model across multiple medical institutions would not only evaluate its scalability but also enrich the training data, potentially leading to improved model accuracy.
- **Broader Medical Imaging Applications**: The success of this model in brain tumor classification opens avenues for applying similar federated learning-based deep learning approaches to other areas of medical imaging, such as detecting tumors in other organs or diagnosing different neurological disorders [27].
- **Model Optimization and Efficiency**: Ongoing research to optimize the model’s computational efficiency and training time can make the approach more feasible for real-world medical settings [28].

In summary, while the federated learning-based CNN model presents a significant improvement in brain tumor classification from MRI images, ongoing research and development are essential to address its current limitations and to explore its full potential in the broader context of medical imaging.

## Conclusion

This study has successfully developed and evaluated a federated learning-based Convolutional Neural Network model for the classification of brain tumors using MRI images. The main findings include the model’s remarkable accuracy rate of 98%, along with high precision and recall across all tumor types: glioma, meningioma, no tumor, and pituitary. These results signify a notable improvement over traditional and existing deep learning methodologies, primarily due to the incorporation of federated learning, which enhances data privacy and model generalization. The study has effectively demonstrated the feasibility and efficacy of using advanced machine learning techniques in the critical domain of medical imaging.

The implications of this research are far-reaching and transformative for the field of medical diagnostics and patient care. The high accuracy and efficiency of the model in classifying brain tumors can significantly aid radiologists and oncologists in making more informed and quicker diagnostic decisions, potentially leading to earlier and more effective treatment plans. This advancement is especially crucial in brain tumor cases, where early detection and accurate classification can markedly influence patient outcomes.

Moreover, the successful application of federated learning in this context opens new avenues for medical data analysis while respecting patient privacy and data security concerns. The model’s approach can be extended to other types of medical imaging tasks, paving the way for broader applications in healthcare diagnostics. It also sets a precedent for future research to explore and refine machine learning models, making them more accessible and practical for clinical use.

In conclusion, this research contributes significantly to the intersection of artificial intelligence and healthcare, showcasing the potential of machine learning to revolutionize medical diagnostics and enhance patient care. However, we recognize the importance of further validation on larger and more diverse datasets to assess the generalizability and robustness of our approach in real-world clinical settings. Additionally, we advocate for exploring extensions of the federated learning framework in other medical imaging tasks beyond brain tumor classification, paving the way for advancements in privacy-preserving AI-driven healthcare solutions. We believe that continued research in this direction will contribute significantly to improving the efficiency, accessibility, and effectiveness of medical imaging technologies, ultimately benefiting patient care and outcomes.

## Data Availability

The datasets used for the findings in the current study are publicly available in the [Kaggle] repository [https://www.kaggle.com/datasets/masoudnickparvar/brain-tumor-mri-dataset].

## References

[1] Islam K, Tohidul S, Wijewickrema, Stephen O’leary (2022). A deep learning framework for segmenting brain tumors using MRI and synthetically generated CT images. Sensors.

[2] Ananth C et al. Blood Cancer Detection with Microscopic Images Using Machine Learning. Machine Learning in Information and Communication Technology: Proceedings of ICICT 2021, SMIT. Singapore: Springer Nature Singapore, 2022. 45–54.

[3] Madhuri G, Sindhu TR, Mahesh, Vivek V. A novel approach for automatic brain tumor detection using machine learning algorithms. Big data management in sensing. River; 2022. pp. 87–101.

[4] Raza A et al. A hybrid deep learning-based approach for brain tumor classification. Electronics 11.7 (2022): 1146.

[5] Wallis D, Buvat Irène (2022). Clever Hans effect found in a widely used brain tumour MRI dataset. Med Image Anal.

[6] Kumar S (2024). A methodical exploration of imaging modalities from dataset to detection through machine learning paradigms in prominent lung disease diagnosis: a review. BMC Med Imaging.

[7] Agrawal T (2024). MultiFeNet: multi-scale feature scaling in deep neural network for the brain tumour classification in MRI images. Int J Imaging Syst Technol.

[8] Zeineldin RA (2022). Explainability of deep neural networks for MRI analysis of brain tumors. Int J Comput Assist Radiol Surg.

[9] Pedada K, Rao (2023). A novel approach for brain tumour detection using deep learning-based technique. Biomed Signal Process Control.

[10] Saeedi S (2023). MRI-based brain tumor detection using convolutional deep learning methods and chosen machine learning techniques. BMC Med Inf Decis Mak.

[11] Mahmud M, Ishtyaq M, Mamun, Abdelgawad A. A deep analysis of brain tumor detection from mr images using deep learning networks. Algorithms 16.4 (2023): 176.

[12] Wang N (2023). Deep learning-based optical coherence tomography image analysis of human brain cancer. Biomedical Opt Express.

[13] Prakash R, Meena (2023). Classification of brain tumours from MR images with an enhanced deep learning approach using densely connected convolutional network. Comput Methods Biomech Biomedical Engineering: Imaging Visualization.

[14] Senan E, Mohammed et al. Early diagnosis of brain tumour mri images using hybrid techniques between deep and machine learning. Computational and Mathematical Methods in Medicine 2022. 10.1155/2022/8330833 PMC9132638 35633922

[15] Haq Aul (2022). DACBT: deep learning approach for classification of brain tumors using MRI data in IoT healthcare environment. Sci Rep.

[16] Rasool M et al. A hybrid deep learning model for brain tumour classification. Entropy 24.6 (2022): 799. 10.3390/e24060799 PMC9222774 35741521

[17] Albalawi E et al. Oral squamous cell carcinoma detection using EfficientNet on histopathological images. Front Med 10 (2023). 10.3389/fmed.2023.1349336 PMC10859441 38348235

[18] Thakur A (2024). Transformative breast Cancer diagnosis using CNNs with optimized ReduceLROnPlateau and Early stopping Enhancements. Int J Comput Intell Syst.

[19] Khan A, Hannan (2022). Intelligent model for brain tumor identification using deep learning. Appl Comput Intell Soft Comput.

[20] Gaur L (2022). Explanation-driven deep learning model for prediction of brain tumour status using MRI image data. Front Genet.

[21] Vidyarthi A (2022). Machine learning assisted methodology for multiclass classification of malignant brain tumors. IEEE Access.

[22] Lamrani D (2022). Brain tumor detection using mri images and convolutional neural network. Int J Adv Comput Sci Appl.

[23] Islam M (2023). Effectiveness of federated learning and CNN ensemble architectures for identifying brain tumors using MRI images. Neural Process Lett.

[24] Alshammari A (2022). Construction of VGG16 convolution neural network (VGG16_CNN) classifier with NestNet-based segmentation paradigm for brain metastasis classification. Sensors.

[25] Chakravarthy S, Nagarajan B, Kumar VV (2024). Breast tumor classification with enhanced transfer learning features and selection using chaotic map-based optimization. Int J Comput Intell Syst.

[26] Mahesh TR, Santhakumar D, Balajee A, Shreenidhi HS, Kumar VV, Rajkumar Annand J. Hybrid Ant Lion Mutated Ant Colony Optimizer Technique With Particle Swarm Optimization for Leukemia Prediction Using Microarray Gene Data, in IEEE Access, vol. 12, pp. 10910–10919, 2024, 10.1109/ACCESS.2024.3351871.

[27] Shivahare B, Dev et al. Medical Image Denoising and Brain Tumor Detection Using CNN and U-Net. 2023 3rd International Conference on Innovative Sustainable Computational Technologies (CISCT). IEEE, 2023.

[28] Asl E, Sima MC, Amirani, Seyedarabi H (2024). Brain tumors segmentation using a hybrid filtering with U-Net architecture in multimodal MRI volumes. Int J Inform Technol.

