# Correction to: Integrated approach of federated learning with transfer learning for classification and diagnosis of brain tumor

**DOI:** 10.1186/s12880-024-01348-8

**Published:** 2024-06-28

**Authors:** Eid Albalawi, Mahesh T.R., Arastu Thakur, V. Vinoth Kumar, Muskan Gupta, Surbhi Bhatia Khan, Ahlam Almusharraf

**Affiliations:** 1 Department of Computer Science, College of Computer Science and Information Technology, King Faisal University, Hofuf, 31982 Saudi Arabia; 2 Department of Computer Science and Engineering, Faculty of Engineering and Technology, JAIN (Deemed-to-be University), Bangalore, 562112 India; 3 School of Computer Science Engineering and Information Systems, Vellore Institute of Technology, Vellore, 632014 India; 4 School of Science, Engineering and Environment, University of Salford, M5 4WT Manchester, UK; 5 Department of Electrical and Computer Engineering, Lebanese American University, Byblos, Lebanon Lebanon; 6 Department of Business Administration, College of Business and Administration, Princess Nourah Bint Abdulrahman University, P.O. Box 84428, Riyadh, 11671 Saudi Arabia

**Correction to: BMC Medical Imaging (2024) 24:110**

10.1186/s12880-024-01261-0

Following the publication of the Original Article, the authors reported that the Funding statement was incorrect.

**Incorrect:**

Princess Nourah bint Abdulrahman University Researchers Supporting Project number (PNURSP2023R432), Princess Nourah bint Abdulrahman University, Riyadh, Saudi Arabia.

**Correct:**

Princess Nourah bint Abdulrahman University Researchers Supporting Project number (PNURSP2024R432), Princess Nourah bint Abdulrahman University, Riyadh, Saudi Arabia.

The original article has been corrected.

